# Alzheimer's disease heterogeneity revealed by neuroanatomical normative modeling

**DOI:** 10.1002/dad2.12559

**Published:** 2024-03-13

**Authors:** Flavia Loreto, Serena Verdi, Seyed Mostafa Kia, Aleksandar Duvnjak, Haneen Hakeem, Anna Fitzgerald, Neva Patel, Johan Lilja, Zarni Win, Richard Perry, Andre F. Marquand, James H. Cole, Paresh Malhotra

**Affiliations:** ^1^ Department of Brain Sciences Faculty of Medicine Imperial College London London UK; ^2^ Centre for Medical Image Computing Medical Physics and Biomedical Engineering University College London London UK; ^3^ Dementia Research Centre UCL Queen Square Institute of Neurology London UK; ^4^ Donders Centre for Cognitive Neuroimaging Donders Institute for Brain Cognition and Behaviour Radboud University Nijmegen The Netherlands; ^5^ Department of Cognitive Neuroscience Radboud University Medical Centre Nijmegen The Netherlands; ^6^ Department of Psychiatry Utrecht University Medical Center Utrecht The Netherlands; ^7^ Department of Nuclear Medicine Imperial College Healthcare NHS Trust London UK; ^8^ Hermes Medical Solutions Stockholm Sweden; ^9^ Department of Neurology Imperial College Healthcare NHS Trust London UK; ^10^ UK Dementia Research Institute Care Research and Technology Centre Imperial College London and the University of Surrey London UK

**Keywords:** Alzheimer's disease, amyloid PET, heterogeneity, MRI, neuroanatomical normative modeling, neurodegeneration

## Abstract

**INTRODUCTION:**

Overlooking the heterogeneity in Alzheimer's disease (AD) may lead to diagnostic delays and failures. Neuroanatomical normative modeling captures individual brain variation and may inform our understanding of individual differences in AD‐related atrophy.

**METHODS:**

We applied neuroanatomical normative modeling to magnetic resonance imaging from a real‐world clinical cohort with confirmed AD (*n* = 86). Regional cortical thickness was compared to a healthy reference cohort (*n* = 33,072) and the number of outlying regions was summed (total outlier count) and mapped at individual‐ and group‐levels.

**RESULTS:**

The superior temporal sulcus contained the highest proportion of outliers (60%). Elsewhere, overlap between patient atrophy patterns was low. Mean total outlier count was higher in patients who were non‐amnestic, at more advanced disease stages, and without depressive symptoms. Amyloid burden was negatively associated with outlier count.

**DISCUSSION:**

Brain atrophy in AD is highly heterogeneous and neuroanatomical normative modeling can be used to explore anatomo‐clinical correlations in individual patients.

## 1 BACKGROUND

For decades, the “typical” Alzheimer's disease (AD) patient has been portrayed as an older adult with marked episodic memory impairment and loss of grey matter volume in the medial temporal lobe (MTL). However, AD can present in several forms, which vary in the age of onset, clinical presentation, and neuropathological and genetic profiles,^1^ and it is a continuum, rather than a series of discrete clinical entities, which goes from normal cognitive status to mild cognitive impairment (MCI) to dementia.^2^, ^3^ Advances in diagnosis, treatment, and understanding of the pathophysiological mechanisms of AD require research to move beyond the idea of a typical AD patient,^4^ as this implies an interindividual homogeneity that is not reflected in the real‐world clinical population. Not all AD patients present with a typical phenotype and age of onset, and failure to recognize this frequently leads to diagnostic delays and errors.^5^

The dominant approach in case‐control studies is to compare the average atrophy patterns in AD patients with those in healthy individuals. While this method enables the important detection of hallmarks of typical AD, such as MTL atrophy,^6^ it provides limited information about the variability of disease mechanisms within this clinical population.^7^ In case‐control studies AD patients are grouped together, hence considered comparable to each other and clearly distinct from healthy controls. This implies an underlying assumption of intragroup homogeneity and defines the disease as a discrete entity rather than as a continuum. Moreover, this approach suggests that typical AD likely represents a more homogeneous group, often made the reference standard in AD research and clinical trials.

Neuroanatomical normative modeling is an emerging statistical technique that differs from the prevailing approach of clustering,^7^ which has been the predominant avenue for the exploration of heterogeneity in dementia to date (see Habes et al. for a review^8^). Neuroanatomical normative modeling shifts the focus from group averages to intracohort variation,^9^ aiming to gather individual‐level information by comparison with extensive datasets of healthy control participants.^7^, ^9^ This is done by estimating centiles of variation of a brain measure (eg, cortical thickness) across the normative population and then assessing how much each individual deviates from the respective distribution.^4^ Moreover, it examines the extent to which an individual deviates from the norm at any given brain region, providing a map of individual variability.^4^ Widely used in psychiatric research over recent years,^10^, ^11^, ^12^, ^13^, ^14^, ^15^, ^16^, ^17^ this technique has had limited use in AD research so far.^18^, ^19^ In a recent application by Verdi and colleagues, neuroanatomical normative models revealed a largely heterogeneous distribution of cortical atrophy in the Alzheimer's Disease Neuroimaging Initiative (ADNI) research cohort.^20^, ^21^

In the present study, for the first time, we applied neuroanatomical normative modeling employing hierarchical Bayesian regression to a real‐world clinical cohort, mapping structural variation in diagnostically challenging patients who required biomarker confirmation of AD. The Imperial Amyloid PET Cohort (APC) was established at Imperial College Healthcare NHS Trust (ICHT) in 2013 and includes all patients seen at the Imperial Memory Clinic and receiving amyloid positron emission tomography (PET) imaging as part of their diagnostic workup^22^ in line with appropriate use criteria.^23^ The objectives of the current study were to (1) assess intragroup neuroanatomical heterogeneity in a real‐world clinical cohort of patients with confirmed AD; (2) explore anatomo‐clinical correlations at an individual level; (3) examine the association between global amyloid burden and deviations in cortical thickness.

## 2 METHODS

### 2.1 Subjects

Of 256 amyloid‐positive patients from the Imperial APC Cohort scanned between 2014 and 2021, we included those who had a clinical magnetic resonance imaging (MRI) scan performed within 12 months of amyloid PET (*n* = 186). Of these, 82 were excluded due to unavailable or ineligible T1‐weighted images, motion artifacts, other pathologies affecting brain integrity (ie, normal pressure hydrocephalus, multiple sclerosis, and large infarcts), or segmentation failure. Of the remainder, 18 were scanned externally and were excluded by the model, leaving a total of 86 patients, hereafter termed the *clinical cohort* (Figure S1A). The *reference cohort* consisted of a group of 33,072 cognitively normal adults pooled from publicly available neuroimaging datasets under a previous study^24^; within this group,  17,586 (53.2%) participants were aged between 49 and 87 years, which was the age range in the clinical cohort studied. The adaptation dataset consisted of a group of 20 cognitively normal (CN) older adults who had an MRI scan at ICHT for research purposes.

### 2.2 MR image acquisition

All subjects had whole‐brain T1‐weighted volumetric images. Of the 104 patients in the clinical cohort, 86 (83%) were scanned at ICHT using a 1.5T Siemens MAGNETOM Avanto (repetition time = 900 ms; echo time = 3.37 ms; 160 slices/slab, voxel size of 1 × 0.5 × 0.5 mm), while the remaining 18 were scanned externally and these were excluded from the model. Participants in the adaptation dataset were scanned at ICHT using a 3T Siemens MAGNETOM Verio (repetition time = 900 ms; echo time = 2.52 ms; 176 slices/slab, voxel size of 1 × 1 × 1 mm). The imaging protocol for the reference cohort is reported in.^20^

### 2.3 MR image analysis

#### 2.3.1 Cortical segmentation

Cortical reconstruction and volumetric segmentation were performed using the FreeSurfer 6.0 recon‐all function (https://surfer.nmr.mgh.harvard.edu/),^25^ as detailed in Figure S2. To compare directly with the reference cohort, we used the Destrieux atlas of 148 cortical parcellations (74 in each hemisphere), classified as gyral or sulcal.^26^ Cortical segmentation procedures for the reference cohort are described elsewhere.^24^

#### 2.3.2 Neuroanatomical normative modeling

Hierarchical Bayesian regression proposed by Kia and colleagues was used, given its advantages over other methods for normative modeling of real‐world clinical data^27^, ^28^ (see Supplementary Appendix 1 for details). Hierarchical Bayesian regression was previously trained on the reference cohort (compiled by Kia and colleagues) consisting of a large sample of healthy controls who did not have any known clinical symptoms at the time of scanning, using age and sex as the covariates to index population variability in cortical thickness across all 148 regions of interest (ROIs) (see Table S1 for cohort details).^7^ This model was then optimized using cortical thickness data from controls scanned at the same acquisition site as the clinical cohort (*n* = 20). This gives stable estimates of the transferred model parameters in an adapted transfer learning approach. This recalibrated model was then used to generate regional cortical thickness z‐scores for each participant in the clinical cohort, relative to the normative range of the reference cohort (Figure S1B). Here, z‐scores of <−1.96 were defined as outliers, representing the bottom 2.5% of the normative range and indicating an extreme negative deviation of cortical thickness. This threshold has been adopted in similar studies to which our outputs can be conceptually compared.^20^, ^21^, ^29^ Analysis of outliers was limited to negative deviations as the primary interest of this study was AD‐related neurodegeneration as indexed by lower cortical thickness. The *total outlier count* was calculated by summing the number of outlier regions for each patient. To assess the spatial distribution of these deviations (ie, areas with marked lower cortical thickness), we built individualized outlier maps. Figure S1B illustrates an overview of our method.

**RESEARCH IN CONTEXT**

1. **Systematic review**: The authors reviewed the literature using traditional (eg, PubMed) sources. Previous work has applied neuroanatomical normative modeling to psychiatry with the aim of conceptualizing disorders as deviations from expected functioning and parsing disease heterogeneity. Only one recent study on the Alzheimer's Disease Neuroimaging Initiative research cohort has applied this technique to Alzheimer's disease.
2. **Interpretation**: This study illustrates the potential of applying neuroanatomical normative modeling to a real‐world clinical cohort. Shifting the focus from group means to intragroup variation in Alzheimer's disease could transform the way this disease is studied, diagnosed, and conceptualized by researchers, with considerable implications for clinical trials.
3. **Future directions**: Future studies are needed to replicate and extend our findings to other clinical cohorts. More granular measures of clinical features such as cognition, affective symptoms, and severity, as well as apolipoprotein E genetic status, will be needed to better understand the factors underlying structural heterogeneity.

### 2.4 Amyloid PET image acquisition

All patients included in this study were scanned using a Siemens Biograph 64 PET/computed tomography (CT) scanner. The ligand changed from 18F‐florbetapir (Amyvid) to 18F‐florbetaben (Neuraceq) in December 2017, following the cessation of 18F‐florbetapir manufacture in the UK. Amyloid PET acquisition for this cohort was as previously described.^30^

### 2.5 Amyloid PET review and analysis

#### 2.5.1 Clinical interpretation

Amyloid PET images were visually read by an expert nuclear medicine radiologist as amyloid‐positive or amyloid‐negative using greyscale images and the cerebellum as the reference region.^31^ Equivocal cases were independently read by two nuclear medicine radiologists and by a third when there was disagreement.

#### 2.5.2 Amyloid quantification

Quantification of amyloid PET images was performed using Hermes BRASS version 4.0 (Hermes Medical Solutions AB, Stockholm, Sweden), a fully automated PET‐only driven method fully described elsewhere.^32^ This method provides a regional standardized uptake value ratio (SUV_R_), computed across 48 ROIs,^32^ and a global amyloid beta (Aβ) index. The SUV_R_ is the ratio between tracer uptake within each ROI to that in the reference region which, in this study, was the cerebellum.^33^ The Aβ index corresponds to the total weight of global amyloid deposition, and it ranges between −1 (Aβ‐negative appearance) and +1 (Aβ‐positive appearance).^32^, ^34^

#### 2.5.3 Clinical measures

To examine how individual deviation profiles related to clinical features, we retrospectively collected patients presenting phenotype (amnestic vs non‐amnestic), disease stage (MCI vs dementia) at the time of MRI, and history of depressive symptoms (Table 1) through a structured review of clinical records. The definition of MCI and depression history adopted in this study are described in Petersen et al. 2004^35^ and in Loreto et al. 2022,^36^ respectively.

**TABLE 1:** Demographic and clinical information of Alzheimer's patients.

	Aβ‐pos (*n* = 86)
**Demographics**	
Mean age ± SD (years)	67.56 ± 8.06
Age range	49.1–87.4
Sex (female), *n* (%)	42 (48.8%)
Presentation (amnestic/non‐amnestic)	64/22
Non‐amnestic	22
Visuospatial, *n* (%)	7 (32%)
Language, *n* (%)	11 (50%)
Behavioral, *n* (%)	4 (18%)
Alzheimer's disease stage (dementia/MCI)	48/38
Depressive symptoms	82
Ongoing, *n* (%)	24 (29%)
Past, *n* (%)	7 (9%)
None, *n* (%)	51 (62%)
**Appropriate use criteria** ^23^	
Indication 1 Persistent or progressive unexplained mild cognitive impairment	51.2%
Indication 2 Dementia with atypical clinical course or etiologically mixed presentation	44.2%
Indication 3 Dementia with early age of onset (age < 65)	39.5%

Abbreviations: Aβ, amyloid beta; MCI, mild cognitive impairment.

### 2.6 Statistical analysis

#### 2.6.1 Standard case‐control comparisons

To test how a standard case‐control approach performs in this clinical cohort as opposed to the neuroanatomical normative modeling approach, we conducted standard case‐control comparisons on a subgroup of patients and a subgroup of CN adults. Cortical thickness extracted using FreeSurfer was compared between age‐ and sex‐matched groups of patients from the Imperial APC clinical cohort (*n* = 79) and CN individuals from the ADNI reference cohort (*n* = 79) (mean age ± SD = 68.69 ± 7.32 years, 68.71 ± 7.23 years, respectively; females: 50% in both groups). Analysis of covariance (ANCOVA), with age and sex as the covariates, was used to compare mean overall thickness. Region‐level comparison was performed using two‐tailed *t*‐tests at each region, adjusting for multiple comparisons using the false discovery rate (FDR).

#### 2.6.2 Total outlier count analysis

The total outlier count ranges between 0 (no mapped regions are outliers) and 148 (all mapped regions are outliers). The distribution of the total outlier count was tested for normality using the Shapiro–Wilk test, which showed positively skewed data. ANCOVAs, with age and sex as covariates, were run to test for the effect of Group (with grouping based on sex, phenotype, disease stage, or depression history) on log‐transformed outlier count data. The Pearson correlation coefficient was used to test for the association between total outlier count and age.

#### 2.6.3 Analysis of spatial distribution of outliers

We mapped outlier regions on the Destrieux atlas to visualize their spread and distribution at the individual‐ and the group‐level. ANOVAs or Mann–Whitney non‐parametric tests were run to investigate the associations between clinical features and percentage of outliers in single ROIs in specified ones, or across all ROIs. Multiple comparisons were Bonferroni corrected. Intragroup dissimilarities in patterns of outliers were quantified using Hamming distance matrices and median Hamming distances were compared between groups (with grouping based on clinical features or disease severity). Furthermore, all 30 (15 in each hemisphere) temporal gyri and sulci of the Destrieux atlas (*temporal*) were grouped separately from the remaining 118 (59 in each hemisphere) extratemporal gyri and sulci (*extratemporal*). The mean percentage of temporal outliers was compared with that of extratemporal outliers using analysis of variance (ANOVA). A two‐way ANOVA was run testing for the interaction between outlier location (temporal vs extratemporal) and phenotype (amnestic vs non‐amnestic).

#### 2.6.4 Exploratory analyses of brain‐phenotype associations

We ran three separate ANCOVAs, covarying for age and sex, to investigate the association between total outlier count and disease severity (MCI vs dementia), disease phenotype (amnestic vs non‐amnestic), or depression history (ongoing vs no symptoms). When disease phenotype and depression history were used as independent variables, disease severity was included as a covariate. The total outlier count was log‐transformed to meet the normality assumption. Outlier maps were built to compare the spatial distribution of outliers between these groups. Hamming distance matrices and median Hamming distances were used to assess intra‐group dissimilarity, and differences in median Hamming distances between groups were assessed using linear regression.

#### 2.6.5 Amyloid quantification

Linear regression analysis was used to test for the association between total outlier count and mean SUV_R_. Outlier maps were compared between patients with higher (high SUV_R_, *n* = 37) and lower (low SUV_R_, *n* = 49) levels of amyloid, defined as an SUV_R_ respectively above or below the group median.

## 3 RESULTS

Clinical and demographic features are provided in Table 1.

### 3.1 Cortical thickness of clinical AD versus ADNI controls

After controlling for age and sex, mean cortical thickness was significantly lower in the clinical cohort (mean ± SD = 2.29 ± 0.13) than in the ADNI control group (mean ± SD = 2.46 ± 0.11; F_(1,154) _= 88.78, *p* < 0.001). Region‐level comparisons adjusted for multiple comparisons highlighted significantly lower thickness in 104 of 148 regions of the clinical cohort (Figure S3).

### 3.2 Total outlier count

The median number of outlier regions in the clinical cohort (*n* = 86) was 21.5 (interquartile range [IQR] = 35) and the total outlier count ranged between 1 and 120. Females had a significantly higher number of outliers (median = 31.5, IQR = 52) than males (median = 17.5, IQR = 33; *U* = 565, *p* = 0.002), while there was no association between age and total outlier count (*r* = −0.17, *p* = 0.11).

### 3.3 Regional distribution of outliers

**FIGURE 1:**
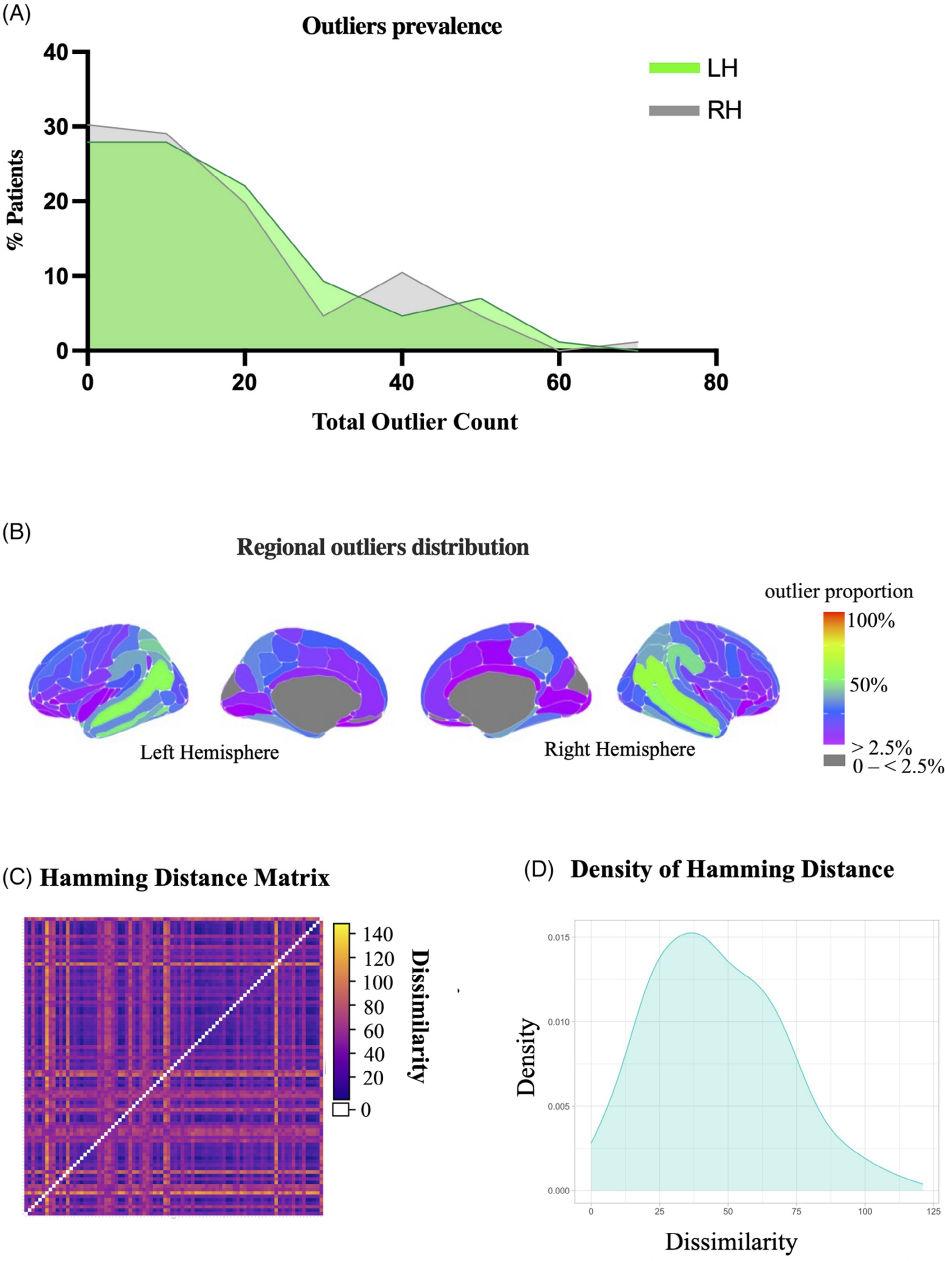
Overall outlier distribution. (A) Distribution of outlier prevalence across the left (LH) and right (RH) hemispheres. (B) Outlier maps showing spatial distribution of outliers in the clinical cohort (*n* = 86). The superior temporal sulci (in green) featured the highest number of outliers (ie, regions with significantly reduced thickness compared to the norm) in both hemispheres. (C) Hamming distance plot illustrating dissimilarity between patients in the spatial distribution of outliers. Yellow indicates greater dissimilarity. (D) Outlier distance density illustrates the spread of outlier dissimilarity (calculated by Hamming distance).

The proportion of outliers was comparable between the left hemisphere (lh; median = 21.5%, IQR = 18%) and right hemisphere (rh; median = 19%, IQR = 17%; *U* = 2620, *p* = 0.65) (Figure 1A). The superior temporal sulcus (STS) featured the highest proportion of outliers in both hemispheres (lh: 52%, rh: 60%) (Figure 1B). Specifically, this was classified as an outlier in both hemispheres in 48% of patients, in either the left or right in 17% of patients, and in none in 35%. Patients with bi‐hemispheric STS outliers had significantly lower mean cortical thickness and younger age than the other two groups and presented with more non‐amnestic symptoms and more advanced disease stages than those with no STS outliers (Table 2). Hamming distance matrices indicated within‐group dissimilarity (Figure 1C,D) (median = 35.25, IQR = 20.75).

**TABLE 2:** Comparison of clinical and demographic characteristics according to STS outlier status.

	No STS outliers (*n* = 30)	Left *or* right STS outliers (*n* = 15)	Left *and* right STS outliers (*n* = 41)	Significance
Mean age ± SD (years)	71.11 ± 6.31^c^	70.35 ± 8.26^c^	63.93 ± 7.71^a^, ^b^	F_(2,83) _= 9.59, *p* < 0.001
Mean cortical thickness ± SD (mm)	2.38 ± 0.093^c^	2.31 ± 0.064^c^	2.21 ± 0.13^a^, ^b^	F_(2,83) _= 21.31, *p* < 0.001
Sex (% female)	30%^d^	66.7%^d^	56.1%	χ^2^_(2) _= 7.03, *p* = 0.03
Disease stage (% dementia)	40%^c^	46.6%	70.7%^a^	χ^2^_(2) _= 7.25, *p* = 0.03
Phenotype (% amnestic)	93.3%^c^	66.7%	34.14%^a^	χ^2^_(2) _= 8.72, *p* = 0.01

*Note*: Bonferroni‐adjusted significance. STS, superior temporal sulcus.

^a^ Significantly different from “No STS outliers.”

^b^ Significantly different from “Left *or* right STS outliers.”

^c^ Significantly different from “Left *and* right STS outliers.”

^d^ Trend towards significance (*p* = 0.057).

#### 3.3.1 Temporal lobe

The mean percentage of temporal outliers was 31.5% (SD = 13.7%), ranging between 7% in the left lingual gyrus and 56% in the STS. This was significantly higher than the extratemporal regions (19.1% ± 10.5%, F_(1,146) _= 29.39, *p* < 0.001), where it ranged from 0% in the left suborbital sulcus to 47% in the right supramarginal gyrus. There was no interaction between outlier location and phenotype (F_(1,144) _= 0.003, *p* = 0.96), suggesting a comparable difference between the proportion of temporal and extratemporal outliers in amnestic (mean difference = 12%) and non‐amnestic (mean difference = 13%) groups.

#### 3.3.2 Disease stage

**FIGURE 2:**
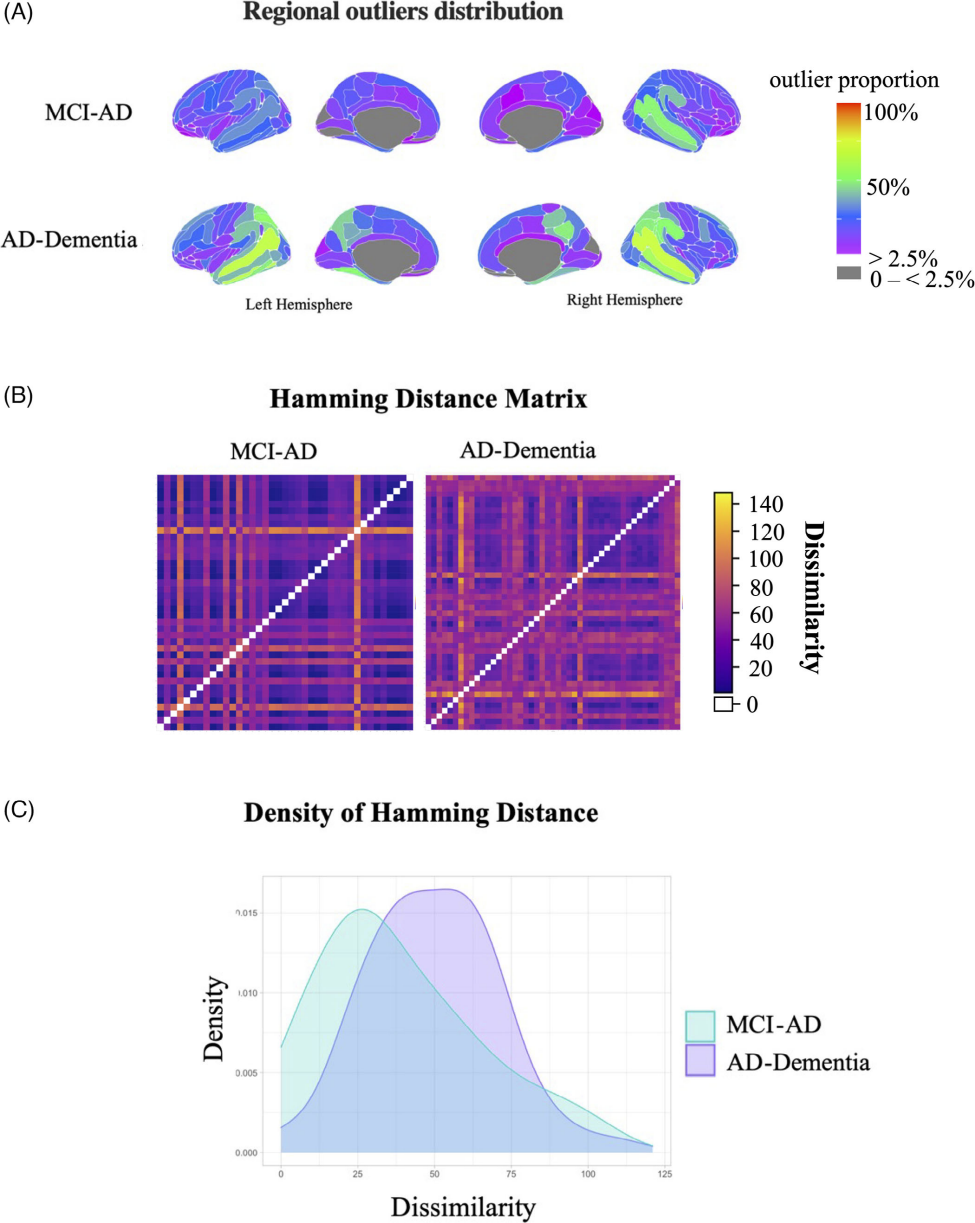
Outlier profiles according to disease severity. (A) Outlier maps showing distribution of outliers according to disease severity. (B) Hamming distance plot illustrating dissimilarity between patients in the spatial distribution of outliers; the yellow color indicates greater dissimilarity. (C) Outlier distance density illustrates the spread of outlier dissimilarity (calculated by Hamming distance). AD, Alzheimer's disease; MCI, mild cognitive impairment.

The total outlier count was significantly higher in the AD‐dementia group (median = 30, range 2 to 120) than in the MCI‐AD group (median = 17.5, range 1 to 109; F_(1,82) _= 8.33, *p* = 0.005). In AD‐dementia, the most frequently outlying region was the STS in both hemispheres (lh: 67%, rh: 69%). In MCI‐AD, the most frequently outlying region was the STS in the right (50%) and the planum polare in the left hemisphere (37%) (Figure 2A).^26^ In both groups, outliers were widespread across the brain with a limited overlap of outlying regions outside the temporal lobe (Figure 2A), suggesting highly heterogeneous patterns of atrophy not explained by disease severity. Greater within‐group dissimilarity (F_(1,82) _= 8.15, *p* < 0.01) was found in the AD‐dementia group (median = 41, IQR = 22) relative to the MCI‐AD group (median = 28, IQR = 21) (Figure 2B,C).

#### 3.3.3 Presenting phenotype

**FIGURE 3:**
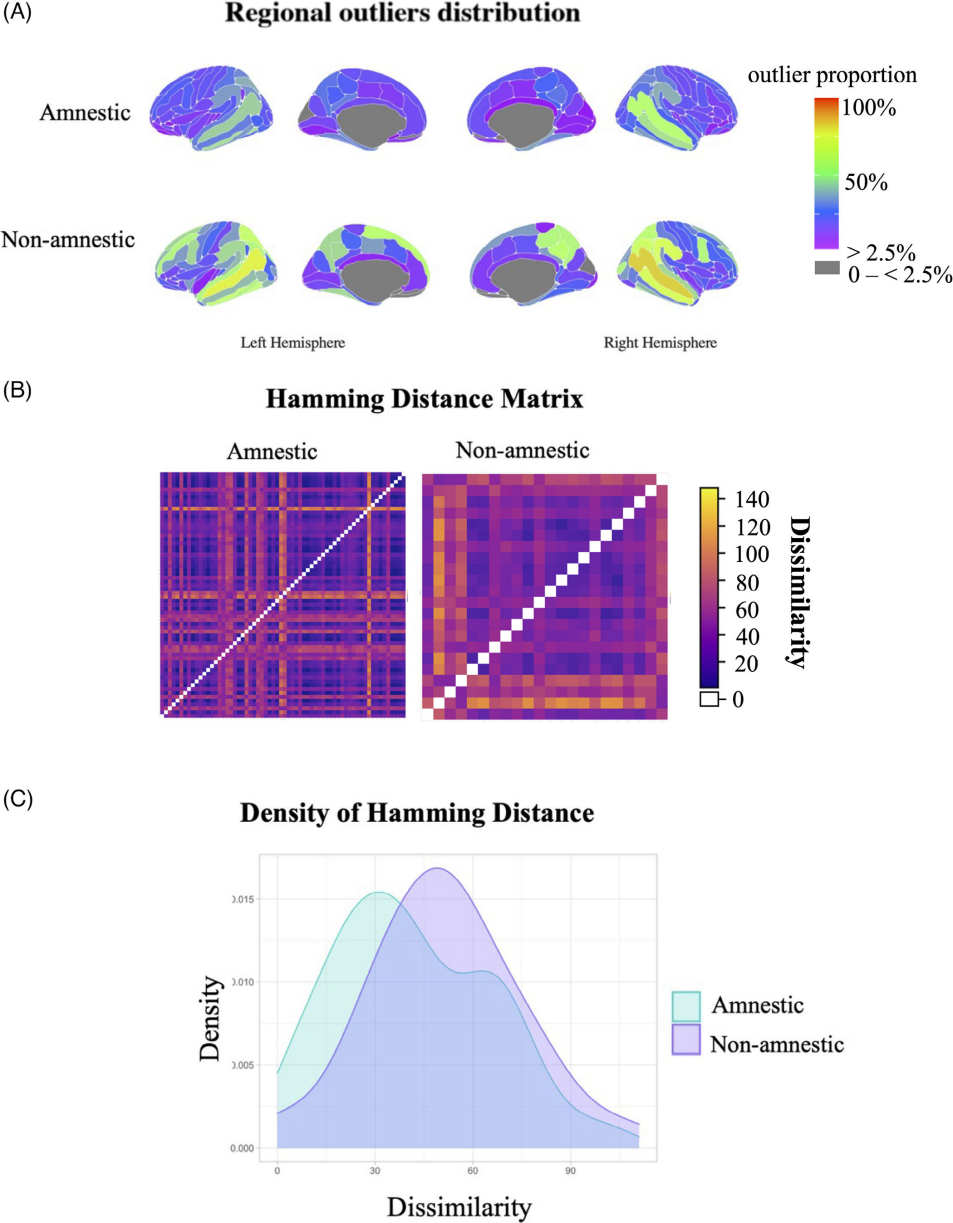
Outlier profiles according to phenotype. (A) Outlier maps showing distribution of outliers according to phenotype. (B) Hamming distance plot illustrating dissimilarity between patients in the spatial distribution of outliers; the yellow color indicates greater dissimilarity. (C) Outlier distance density illustrates the spread of outlier dissimilarity (calculated by Hamming distance).

The total outlier count was significantly higher in the non‐amnestic (median = 37.5, range 11–120) than in the amnestic (median = 19.5, range 1–109) group (F_(1,81) _= 5.49, *p* = 0.02). In the amnestic group, the most frequently outlying region was the STS in the right hemisphere (53%) and the inferior temporal sulcus in the left hemisphere (47%). In the non‐amnestic group, the STS was the most frequently outlier in both hemispheres (lh: 77%, rh: 82%) (Figure 3A). Greater within‐group dissimilarity (F_(1,84) _= 8.13, *p* < 0.01) was found in the non‐amnestic group (median = 44.75, IQR = 15.38) than in the amnestic group (median = 31.5, IQR = 19.62) (Figure 3B,C).

#### 3.3.4 Comorbid depressive symptoms

The total outlier count was significantly higher in patients without a history of depression (median = 30, IQR = 47) than in those with ongoing depression (median = 16, IQR = 15; F_(1,70) _= 8.56, *p* = 0.005). The STS was the most frequently outlying region in both hemispheres in patients without (lh: 59%, rh: 65%) and with (lh: 42%, rh: 50%) ongoing depression. Greater within‐group dissimilarity (F_(1, 73) _= 24.69, *p* < 0.001) was found in patients without depression (median = 42, IQR = 24) than in those with ongoing depression (median = 25.5, IQR = 8.8).

### 3.4 Case series

**FIGURE 4:**
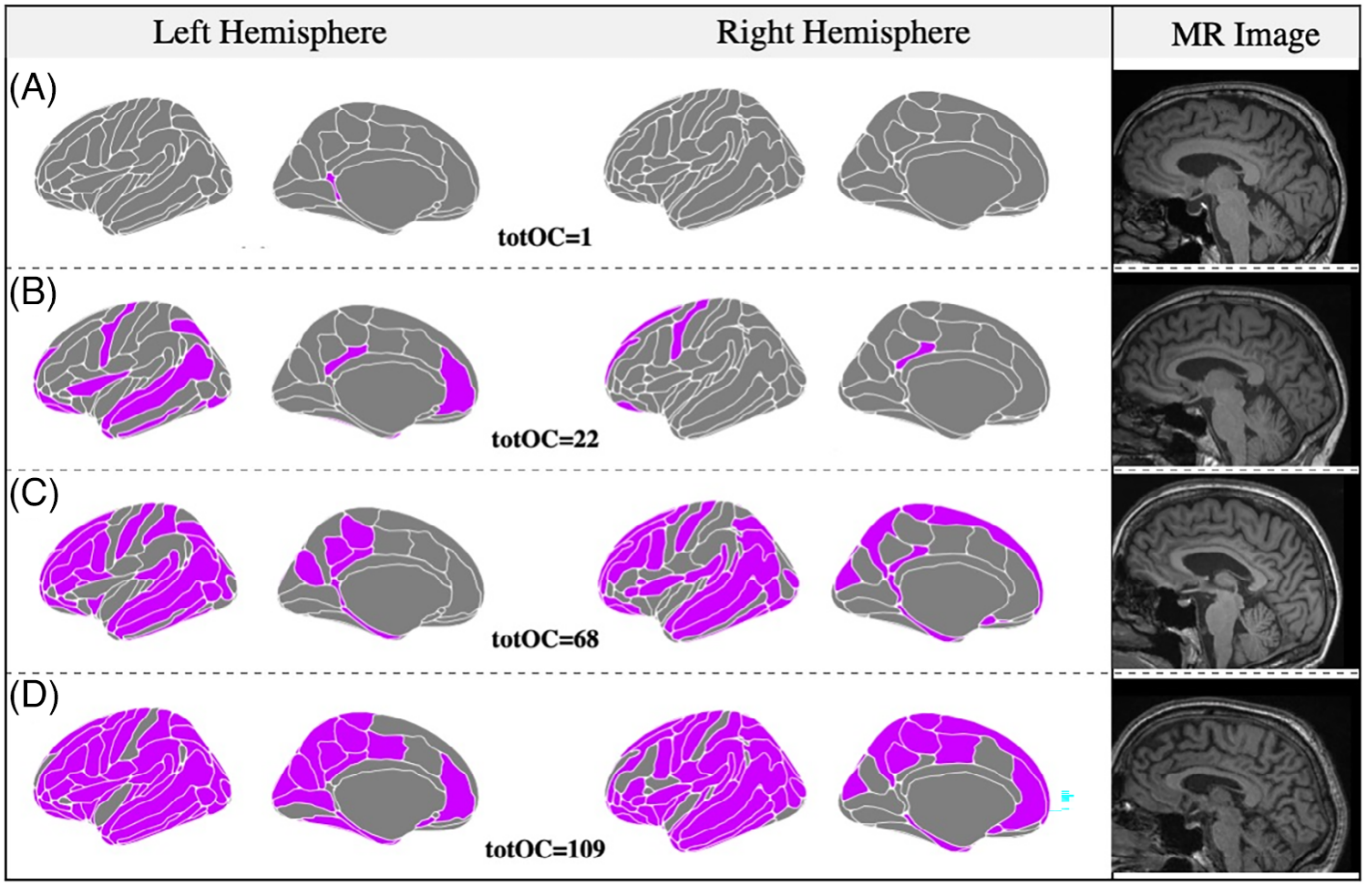
Case series. This short case series illustrates the possible use of outlier maps to gain insight into the association between atrophy profiles and clinical history. These four MCI patients had a similar clinical presentation, a positive amyloid PET imaging, but very heterogeneous patterns of outlier regions. Purple‐colored areas indicate outlier regions (z‐score < 1.96). This finding corroborates the large heterogeneity of AD atrophy profiles at presentation and indicates another possible application of normative modeling for a closer investigation of anatomo‐clinical associations. (A) A man in his 70s presenting to our clinic with a 3‐year history of memory problems, intact activities of daily living (ADLs) and preserved insight. Medical history review did not highlight significant comorbidities or depressive symptoms. On examination, he scored 94/100 on the ACE‐III and 26/30 on the MMSE. Clinical follow‐up revealed a slow progression of cognitive deficits. (B) A man in his late 60s presenting with a 4‐year history of memory problems and preserved ADLs. Insight into the cognitive difficulties was limited and collateral account reported behavioral features such as passivity and reduced empathy. No history of depression was recorded. On examination, ACE‐III score was 85/100. Follow‐up visits revealed slow progression of the cognitive deficits with relative sparing of ADLs. (C) A lady in her 70s presenting with a 2‐year history of memory problems with intact ADLs, preserved insight, and no history of depression. MMSE score was 26/30. Follow‐up visits revealed a steady decline with gradual involvement of ADLs. (D) A man in his mid‐60s presenting with a 2‐year history of memory problems and intact ADLs and no history of depression. The ACE‐III score was 78/100 and follow‐up visits highlighted clinical progression. The MMSE score at 2 years following the first examination was 22/30. ACE‐III, Addenbrooke's Cognitive Examination version III^37^; AD, Alzheimer's disease; ADLs, activities of daily living; MCI, mild cognitive impairment; MMSE, mini‐mental state examination^38^; MR, magnetic resonance; PET, positron emission tomography; totOC, total outlier count.

An important potential use of normative modeling framework in AD involves the investigation of how individual profiles of deviations relate to the clinical presentation and course of the disease. This allows a closer investigation of anatomo‐clinical associations at the individual level while parsing disease heterogeneity. An example of this practical application of normative modeling is provided in Figure 4. This is a short case series of four patients selected from the clinical cohort who presented to our clinic with comparable clinical features but very heterogeneous outlier profiles. For these patients, we collected further information from the clinical records, including the clinical picture at the time of presentation to our clinic, level of cognitive impairment at screening (as measured by the Addenbrooke's Cognitive Examination [ACE] and/or the Mini‐Mental State Examination [MMSE]), and course of the disease over clinical follow‐ups. Further details on a case‐by‐case basis are provided in the legend of Figure 4.

### 3.5 Association between total outlier count and amyloid burden

Mean SUV_R_ was negatively associated with total outlier count (*p* = 0.01, *R* ^2 ^= 0.077) and positively with raw mean cortical thickness (*p* = 0.01, *R* ^2 ^= 0.08) (Figure S4). Both associations survived after controlling for age (*p* = 0.02, *R* ^2 ^= 0.093 for total outlier count;  *p* = 0.03, *R* ^2 ^= 0.082, for mean cortical thickness). The lowest mean regional SUV_R_ was in the anterior division of the parahippocampal gyrus (1.08 ± 0.15), while the highest was in the posterior division of the cingulate gyrus (1.99 ± 0.31). Notably, patients classified in the low SUV_R_ group (ie, individual mean SUV_R_ value < group median) showed a higher number of outlying regions and higher within‐group dissimilarity than those classified in the high SUV_R_ group (Figure S5).

## 4 DISCUSSION

In this study, we applied normative modeling to a real‐world clinical cohort with confirmed AD and found that the total outlier count varied widely across patients. The individual magnitude of deviation ranged between 1 and 120 out of 148 ROIs (median 21.5). Outlier maps revealed prominent involvement of the superior temporal sulci, which were affected in up to 60% of patients, most frequently in younger and non‐amnestic patients. Our findings are in line with those reported by Verdi et al. on the ADNI cohort in which the STS was among the set of temporal outlier regions differentiating AD from MCI and controls. On the other hand, in Verdi et al.'s study, the STS was an outlier in about one‐third of patients (36% and 31% in the left and right hemispheres respectively) and the highest proportion of outliers was found in the left parahippocampal gyrus (47%).^20^ In the present clinical cohort, the left parahippocampal gyrus was classified as an outlier in 30% of all subjects and 31% of the AD‐dementia subgroup. Differences in the outlier maps between the two studies may be due to different clinical features as well as cohort types, given that the ADNI study is solely based on clinical criteria and did not involve biomarker confirmation of AD (http://adni.loni.usc.edu). Moreover, patients meeting appropriate use criteria for amyloid PET are, by their very nature, more likely to present with so‐called atypical features.^23^ As such, the study of this cohort provides insight into AD heterogeneity and the potential limitations of standard diagnostic approaches, which are based on the assumption of disease homogeneity. Notably, we found that no brain region deviated in more than 52 out of 86 clinical patients with confirmed Alzheimer's pathology. Furthermore, a relatively large proportion of patients did not significantly deviate from the norm in any of the temporal regions, despite amnestic presentation. These findings bring the ongoing validity of a “typical Alzheimer's disease patient” into question.

We broadly characterized the presenting clinical picture of our cohort to explore anatomo‐clinical associations using normative models for the first time in AD. The AD‐dementia group showed a significantly higher outlier count and higher dissimilarity in the regional distribution of outliers. This reflects the expected greater involvement of cortical areas as disease progresses.^39^ With respect to disease phenotype, the non‐amnestic group showed a significantly higher total outlier count and greater within‐group dissimilarity than the amnestic one. This was not surprising as the non‐amnestic group would have encompassed a wider range of phenotypes, each with prominent involvement of different networks of brain regions.^1^ The presence of concomitant depressive symptoms was associated with a lower mean outlier count and reduced within‐group dissimilarity. A recent study reported a significant association between the severity of depressive symptoms and STS thickness in a group of patients with clinical AD.^40^ In our cohort, the average proportion of outliers in this region was indeed high but comparable between patients with (46%) and without (62%) depression. We did not identify any cortical regions selectively involved in patients with depression, although a different pattern may have been revealed by the analysis of subcortical structures.^41^

The negative association between outlier count and SUV_R_ was an unexpected finding as this would suggest higher cortical volumes in patients with increased burden of amyloid pathology. This was corroborated by the significant positive association between SUV_R_ and raw mean cortical thickness. It is possible that, within the group of amyloid‐positive patients, the SUV_R_ starts decreasing with decreasing cortical volumes or that this relationship is related to the assumptions required for automated SUV_R_ calculation. An important future step of this work will be the assessment of how the regional distribution of outliers aligns with regional variations in SUV_R._

Our rationale for using the threshold of the clinical z‐scores (z < 1.96) was to design a singular marker of individualized heterogeneity at the regional level and across regions.^42^ We believe that the exploration of such markers will have better translational value in clinical settings for aiding personalized decision‐making as they may be easily interpreted as a standardized measure of atrophy outliers. However, future studies could map out disease heterogeneity at the regional level using the full range of z‐scores, which would therefore not exclude patients with scores close to this threshold. The neuroanatomical normative model method employed in this study treats brain regions independently by running separate models for different brain regions. However, it is important to note that regions are related in terms of structural covariance across the brain, which should be considered when interpreting the Hamming distances reported in this study and the brain outlier maps. Future normative modeling studies could therefore also explore how outliers generated for each region are intercorrelated, particularly between neighbouring or bilateral regions. Possible solutions to better understand this include considering the spatial extent and magnitude of affected voxels^43^ or using normative models that incorporate brain connectivity data, which have recently shown promising results.^44^

This study's limitations include the relatively small sample size, partly due to the unavailability of eligible T1‐w data in clinically acquired scans. Moreover, while scanning was conducted at the same site, there was scanner and field strength mismatch between the clinical and adaptation datasets, which may contribute to unwanted noise in the model. As sourcing both scanner‐ and site‐matched controls may be difficult in real‐world clinical studies (as opposed to typical research cohorts), future studies should explore the effects of different scanner strengths on the model output. In this study, strict criteria were adopted at the time of image selection and at output evaluation to limit unwanted noise and ensure that the observed outliers represent clinically relevant deviations (rather than deviations based on image artefacts or inaccurate segmentation). The retrospective nature of data collection meant that we could not gather granular quantification of cognitive functioning and depressive symptoms. Future studies are required to map out these relationships in addition to understanding how different pathogenic mechanisms, such as apolipoprotein E (*APOE*) genotype or co‐pathologies such as vascular disease, might influence the outlier distribution and the heterogeneity observed in this clinical population. Finally, a comprehensive assessment of the association between atrophy and depression was limited by the unavailability of subcortical outliers, which are currently not part of our normative model.

## 5 CONCLUSION

This study illustrates the possible applications of neuroanatomical normative models to parse neuroanatomical heterogeneity in a real‐world clinical cohort with confirmed neurodegeneration due to AD. Our findings highlight striking variability across patients despite comparable disease stages and presentations. The standard case‐control approach would have hidden the intragroup variation that we were able to observe using neuroanatomical normative modeling, as shown by our standard case‐control comparisons on a subgroup of subjects. As AD research finds its path to precision medicine, it is crucial to incorporate novel methods of analysis that are as free as possible from the assumption of intragroup homogeneity. Neuroanatomical normative modeling provides a systematic approach bridging big data analytics and personalized medicine by shifting the analytical focus from group means to intragroup variation via analysis of individual deviations.^7^, ^9^

## CONFLICT OF INTEREST STATEMENT

J.L. is employed by Hermes Medical Solutions and obtains a salary from them; he is Vice President of Research and Development at Hermes Medical Solutions. Z.W. previously participated in the Eli Lilly PET advisory board and was an amyloid‐PET read trainer. R.P. previously sat on an advisory board for Eli Lilly and received support from GE for research imaging from 2014 to 2018. PM gave an educational talk at a meeting organized by GE. None of the authors currently have funding or support from any commercial organization involved in amyloid PET imaging. Author disclosures are available in the supporting information.

## CONSENT

Not required.

## Supporting information

Supporting Information

Supporting Information

## Data Availability

Data not provided in the article are available upon reasonable request.
