# Supplementary material for: Alzheimer's disease heterogeneity revealed by neuroanatomical normative modeling
Source: Alzheimers Dement (Amst). 2024 Mar 13;16(1):e12559. doi: 10.1002/dad2.12559 (PMC10937817; doi:10.1002/dad2.12559)
Supplement: Supplementary file 1 — Supporting Information [file DAD2-16-e12559-s002.docx]

**Supplementary material**

*
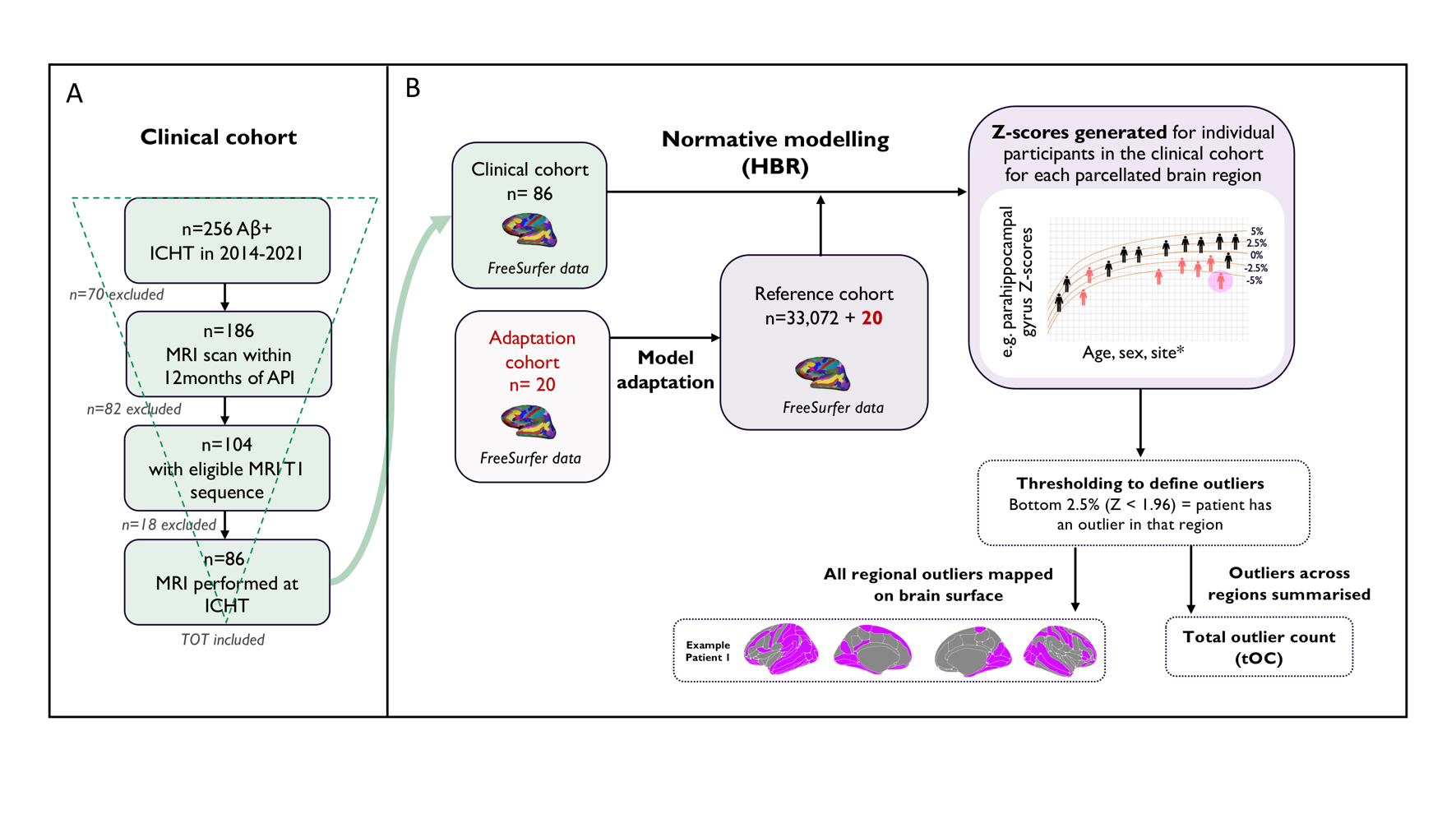
*

**Supplementary Fig.1** **(A)** Flowchart illustrating the number of patients in the clinical cohort that had to be excluded in each stage in line with our inclusion/exclusion criteria. From a total of 256 patients reviewed, 86 were included in the final analysis. **(B)** Schematic diagram illustrating the spatial normative modelling pipeline. Using a separate large reference dataset of healthy participants across the lifespan (‘reference cohort’, n=33,072), neuroanatomical normative models of cortical thickness for separate regions are statistically modelled based on age and sex. The parameters of these models are calibrated using cortical thickness derived from healthy participants that were scanned in the same site as the clinical dataset under investigation (‘adaptation dataset’, n=20). From this, Z-scores relative to the normative range are generated for each brain region for each participant in the real-world clinical sample (‘clinical cohort’, n=86). Outliers in terms of low cortical thickness are identified for each region, which is specifically defined as Z < -1.96 (corresponding to the bottom 2.5% of the normative distribution) – this lower bound threshold for outliers reflects atrophy associated with neurodegeneration. These regional outliers are then mapped onto a brain surface for statistical interpretation and summarised across the brain to give the total outlier count (tOC).


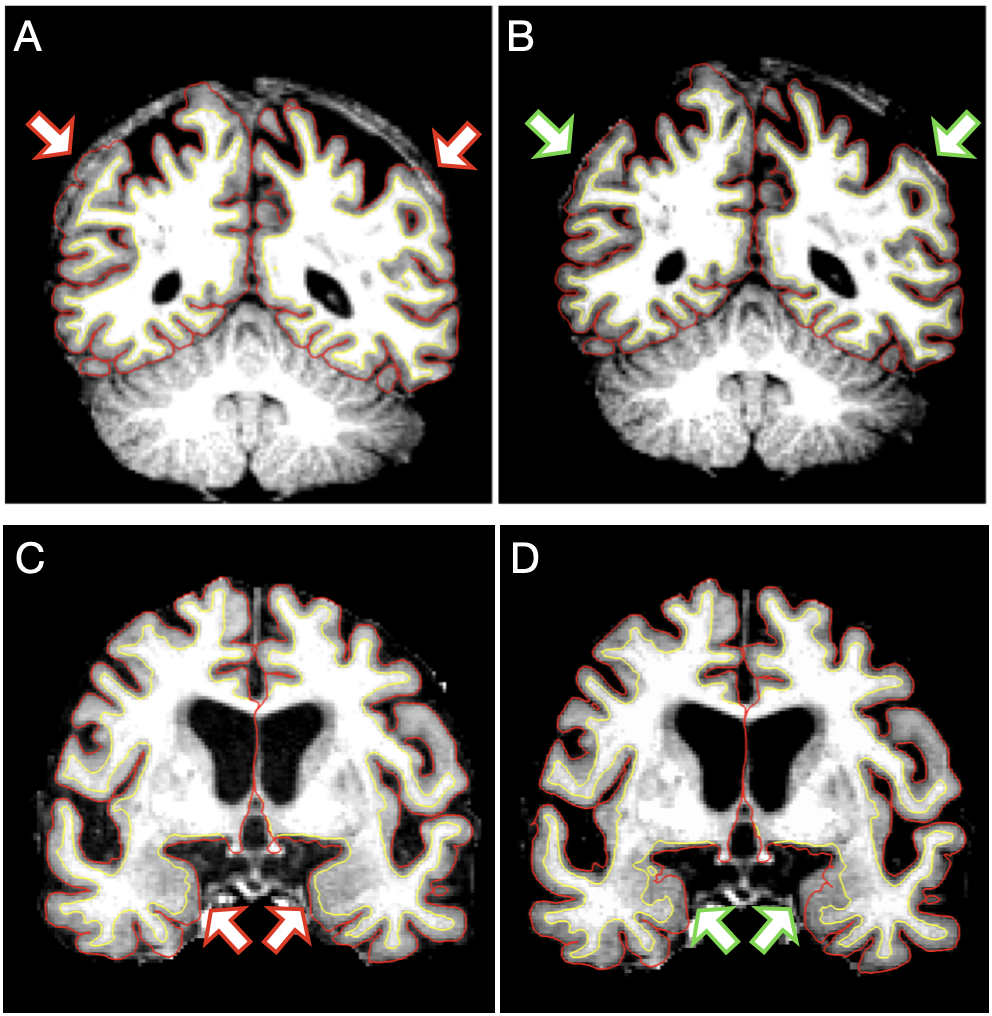


**Supplementary Fig.2** The T1-weighted images of the *clinical* and *validation cohorts*, the output was visually checked for quality control by three authors independently (F.L., A.D., H.H.) following preliminary training on a subset of cases to ensure consistency. Unclear issues were discussed and addressed jointly by the team. Output errors were found in 87 (82%) patients and 19 (68%) CN individuals and addressed in line with FreeSurfer guidelines (<https://surfer.nmr.mgh.harvard.edu/fswiki/FsTutorial/TroubleshootingData>). Most errors occurred in the skull stripping step (75%) leading to the erroneous inclusion of portions of dura or skull within the pial surface as shown in the example (**A**). This type of error was fixed by adjusting input parameters to the skull stripping step and/or by manually editing the brain mask (B). Failure to identify and address this error would have led to the overestimation of parietal volumes in this subject. The remaining 25% of errors occurred at the intensity normalization step (i.e., the homogenization of the signal intensity of the white and grey matter aimed at better differentiation between tissue types) leading to the exclusion of WM and/or GM voxels from the segmented surface or the inclusion of non-WM voxels in the WM surface. The latter case in shown in this example (**C**). This type of error was fixed by adding control points to adjust voxel intensity and/or by manually adding/erasing WM voxels. Failure to identify and address this error would have led to the overestimation of temporal lobe volumes in this subject. After correction, the *recon-all* command was re-run to re-generate surfaces and the output underwent further visual inspection. At this stage, 2 patients had to be excluded due to persistent segmentation errors after multiple correction attempts, leaving a total of 104 patients.


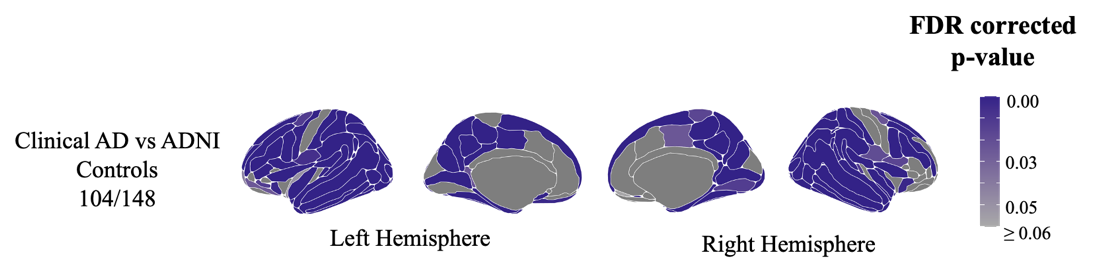


**Supplementary Fig.3.** P-value maps to illustrate significant group differences of cortical thickness at each region.


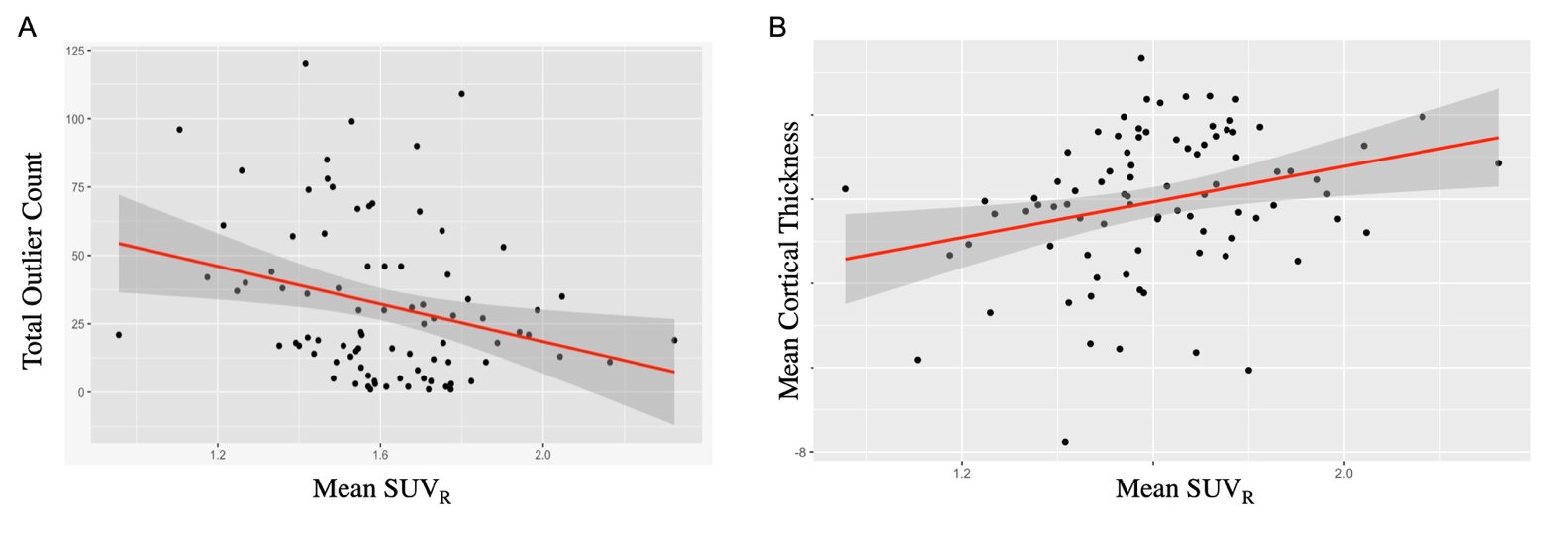
**Supplementary Fig.4** **(A)** Scatter plot showing the association between total outlier count and mean cortical SUV_R_ within the AD clinical cohort. **(B)** Scatter plot showing the association between mean cortical thickness and mean cortical SUV_R_ within the AD clinical cohort. Note: lower outlier count indicates higher cortical thickness.


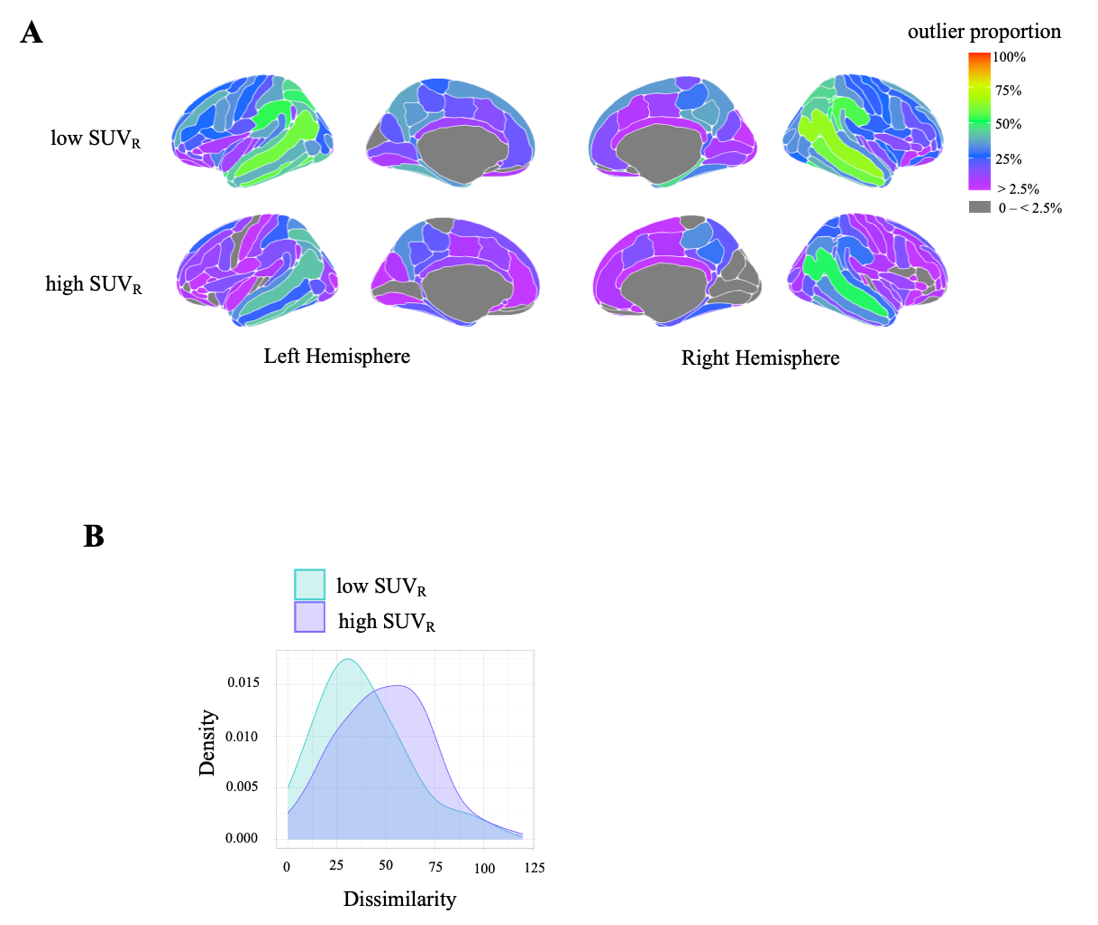


**Supplementary Fig.5** Outlier maps showing distribution of outliers according to **(A)** low or high SUV_R_. **(B)** Outlier distance density illustrates the spread of outlier dissimilarity (calculated by Hamming distance).

**Supplementary Table 1** Demographic breakdown of the datasets which were collated to make the reference dataset.

| **Datasets** | **No. Scans** | **No. Sites** | **Age Range** | **Gender M/F** | **FS Version** | |
| --- | --- | --- | --- | --- | --- | --- |
| **ABCD** | 10732 | 29 | 9-11 | 5589/5143 | 6.0 |  |
| **Cam-CAN** | 647 | 1 | 18-88 | 318/329 | 6.0 |  |
| **CNP** | 264 | 2 | 21-50 | 152/112 | 6.0 |  |
| **FCON** | 1021 | 18 | 8-85 | 439/582 | 6.0 |  |
| **HCP** | 1113 | 1 | 22-37 | 507/606 | 5.3 |  |
| **OASIS3** | 2044 | 5 | 43-97 | 866/1178 | 5.3 |  |
| **PNC** | 1514 | 1 | 8-23 | 731/783 | 6.0 |  |
| **TOP** | 823 | 1 | 17-69 | 435/388 | 6.0 |  |
| **UKBB** | 14914 | 2 | 44-80 | 7133/7781 | 6.0 |  |
| **Total** | 33,072 | 60 | 8-97 | 16170/16902 | - |  |

FS = FreeSurfer**,** ABCD = Adolescent Brain Cognitive Development, Cam-CAN = The Cambridge Centre for Ageing and Neuroscience, CNP = The Consortium for Neuropsychiatric Phenomics, FCON = Functional Connectomes Project, HCP = Human Connectome Project, OASIS3 = Open Access Series of Imaging Studies, PNC = The Philadelphia Neurodevelopmental Cohort, TOP (Norwegian datasets), UKBB = UK Biobank

**Appendix 1. Hierarchical Bayesian Regression for Multi-Site Normative Modelling**

In this study, we have employed a partial pooling methodology, as introduced by Kia et al. (2022), rooted in hierarchical Bayesian regression (HBR) for the purpose of multi-site normative modelling. This approach aims to overcome the limitations associated with conventional techniques, offering enhanced adaptability, stability, and privacy preservation during the development and deployment of normative models.

Multi-site normative modelling entails the examination of neuroimaging data obtained from diverse participant groups across various imaging facilities. These groups may encompass variables such as batch effects, stemming from variations in data collection procedures, or biologically relevant factors like gender and ethnicity, which can sometimes correlate with batch effects. Traditional approaches to normative modeling in the context of multi-site data encompass four strategies: native pooling, pooling following data harmonization, pooling with batch effects treated as fixed factors, and no pooling. Each of these methods presents its own set of limitations, as discussed in detail in Kia et al. (2022).

HBR is an effective means of addressing these limitations, primarily because it incorporates structural dependencies among model parameters by sharing a common prior distribution. In the context of HBR, it is assumed that parameters specific to each data collection site, which govern the data generation process, are drawn from the same prior distribution. To implement this assumption, a wide Gaussian distribution is employed as a weakly informative hyperprior over the parameters associated with the priors. This choice is advantageous as it strikes a balance between the flexibility of the model and computational efficiency, simultaneously acting as a regularization mechanism to prevent overfitting, particularly on small datasets. HBR further enables a compromise between two extreme scenarios: complete pooling and no pooling. This approach fosters the development of more robust models while still allowing for the estimation of distinct parameters for each batch or group. Furthermore, it calculates z-statistics to account for both additive and multiplicative batch effects, obviating the need for data harmonization.

One of the most notable attributes of HBR is its capability to facilitate the transfer of knowledge pertaining to hyperparameters from a reference normative model to secondary datasets, as elucidated in Kia et al. (2022). This transfer learning strategy enables the adaptation of the model to locally collected data without necessitating the sharing of primary data, thus ensuring privacy preservation in federated learning settings. In our study, we have harnessed this feature of the HBR model to transfer the parameters from a reference normative model to our local dataset, demonstrating its practical utility.
